# Health-related quality of life and its predictors among adults living with HIV/AIDS and receiving antiretroviral therapy in Pakistan

**DOI:** 10.1007/s11136-021-02771-y

**Published:** 2021-02-13

**Authors:** Ali Ahmed, Muhammad Saqlain, Naila Bashir, Juman Dujaili, Furqan Hashmi, Faizan Mazhar, Amjad Khan, Musarat Jabeen, Ali Blebil, Ahmed Awaisu

**Affiliations:** 1 School of Pharmacy, Monash University, Jalan Lagoon Selatan, Bandar Sunway, 47500 Subang Jaya, Selangor Malaysia; 2 Department of Pharmacy, Quaid I Azam University Islamabad, Islamabad, Pakistan; 3 HIV Treatment Center, Pims, National AIDs Control Programme, Islamabad, Pakistan; 4 University College of Pharmacy, University of the Punjab, Allama Iqbal Campus, Lahore, 54000 Pakistan; 5 Unit of Clinical Pharmacology, Department of Biomedical and Clinical Sciences L. Sacco, “Luigi Sacco” University Hospital, Università Di Milano, 20157 Milan, Italy; 6 Department of Pharmacy, Quaid-I-Azam university, Islamabad, Pakistan; 7 HIV Centre PIMS Hospital, Islamabad, Pakistan; 8 Department of Clinical Pharmacy & Practice, College of Pharmacy, QU Health, Qatar University, P.O. Box 2713, Doha, Qatar

**Keywords:** Pakistan, Health-related quality of life, HIV/AIDS, EQ-5D-3L, Antiretroviral therapy, Predictors

## Abstract

**Background:**

Health-related quality of life (HRQoL) is considered to be the fourth 90 of UNAIDS 90-90-90 target to monitor the effects of combination antiretroviral therapy (ART). ART has significantly increased the life expectancy of people living with HIV/AIDS (PLWHA). However, the impact of chronic infection on HRQoL remains unclear, while factors influencing the HRQoL may vary from one country to another. The current study aimed to assess HRQoL and its associated factors among PLWHA receiving ART in Pakistan.

**Methods:**

A cross-sectional descriptive study was conducted among PLWHA attending an ART centre of a tertiary care hospital in Islamabad, Pakistan. HRQoL was assessed using a validated Urdu version of EuroQol 5 dimensions 3 level (EQ-5D-3L) and its Visual Analogue Scale (EQ-VAS).

**Results:**

Of the 602 patients included in the analyses, 59.5% (*n* = 358) reported no impairment in self-care, while 63.1% (*n* = 380) were extremely anxious/depressed. The overall mean EQ-5D utility score and visual analogue scale (EQ-VAS) score were 0.388 (SD: 0.41) and 66.20 (SD: 17.22), respectively. Multivariate linear regression analysis revealed that the factors significantly associated with HRQoL were: female gender; age  > 50 years; having primary and secondary education;  > 1 year since HIV diagnosis; HIV serostatus AIDS-converted; higher CD 4 T lymphocytes count; detectable viral load; and increased time to ART.

**Conclusions:**

The current findings have shown that PLWHA in Pakistan adherent to ART had a good overall HRQoL, though with significantly higher depression. Some of the factors identified are amenable to institution-based interventions while mitigating depression to enhance the HRQoL of PLWHA in Pakistan. The HRQoL determined in this study could be useful for future economic evaluation studies for ART and in designing future interventions.

## Background

Low and middle-income countries (LMIC) like Pakistan experience relatively high rates of new human immunodeficiency virus (HIV) infections [1, 2]. Pakistan’s progress towards The Joint United Nations Programme on HIV/AIDS (UNAIDS)’ 90-90-90 goal is lagging behind as only 14% of people living with human immunodeficiency virus and acquired immunodeficiency syndrome (HIV/AIDS) (PLWHA) are aware of their HIV status, only 10% are on treatment, while data on viral suppression are unavailable [3]. Since the advent of safe and effective antiretroviral therapy (ART), AIDS has transformed from a terminal disease to a manageable chronic disease [4, 5]. The use of Highly Active Antiretroviral Therapy (HAART) in PLWHA results in viral load (VL) suppression [6], boosts the immune system [7], and contributes to improving their health-related quality of life (HRQoL) [8]. A new “beyond viral suppression” model proposed to add a “fourth 90” to the UNAIDS’ 90-90-90 target, is to ensure that 90% of PLWHA with VL suppression have good HRQoL [9, 10].

HRQoL is a multidimensional concept [11] that includes the level of independence, physical health, spiritual health, social health, psychological health, and environmental health [12]. The relationship between HIV/AIDS, ART and HRQoL is a complex one [13]. ART helps to prevent the spread of the disease and results in improved QoL and the well-being of PLWHA, but the continuous use of ART required to sustain viral suppression can lead to adverse effects such as lipodystrophy, diarrhoea, and other drug-related symptoms, which have also been shown to affect HRQoL [14, 15]. Several other factors such as age [16], gender [17], socioeconomic factors [8], VL [10, 18], CD4 T lymphocytes count [19], education [8], depression [20, 21], comorbidities [22], stigma and discrimination [23], mental health [24], behavioural factors [22] and poor adherence to ART [25], have been reported to influence HRQoL in PLWHA. However, the factors influencing HRQoL among PLWHA may vary from one setting or country to another and the development of interventions to specific patients’ population should take these differences into consideration.

HRQoL is an important measure that enables healthcare workers to understand patients' perceived satisfaction and perception of illness [26]. Its assessment helps to monitor interventions and develop a variety of health strategies to improve the overall health of the community [11, 27]. In particular, HRQoL of PLWHA receiving ART is essential for monitoring the impact of drug therapy on the progression of the disease [28, 29]. To the best of our knowledge, there was no study conducted to assess HRQoL and the factors influencing it among PLWHA in Pakistan. To date, no HRQoL study has been conducted in Pakistan and therefore a baseline study is deemed necessary to inform health authorities in designing evidence-based interventions that are culturally-sensitive and customized to PLWHA in Pakistan. Generic tools such as the EuroQol five dimension (EQ-5D) scale, World Health Organization Quality of Life (WHOQOL) instrument, and 36-Item Short Form Health Survey (SF-36) questionnaire, as well as disease-specific tools such as Medical Outcomes Study HIV Health Survey (MOS-HIV), short version of WHOQOL-HIV (WHOQOL HIV Bref) have been used to measure HRQoL in HIV patients [11, 29]. In the present study, the generic EQ-5D three-level (EQ-5D-3L) questionnaire was used to permit a comparison of HRQoL across different disease conditions and sub-populations [30]. Another advantage of the EQ-5D is that it generates health utility scores that can be used in economic evaluation studies as well [8, 31]. Thus, this study aimed to: (1) evaluate HRQoL in PLWHA receiving ART; (2) determine the sensitivity of the EuroQol questionnaire to differentiate the HRQoL in different stages of HIV infection, CD4 T lymphocytes, and VL and; (3) identify factors influencing HRQoL in Pakistani patients with diverse cultural background.

## Method

### Study design and setting

This was a cross-sectional study using a validated generic HRQoL questionnaire. The study was conducted from July 2019 to September 2019 at the ART Centre in PIMS Hospital, a 947-bed hospital located in Islamabad Capital Territory (ICT), Pakistan. The ART Centre at PIMS is the largest centre of HIV referral cases in Pakistan, serving more than 4200 registered PLWHA who receive free-of-charge HIV treatment. Approximately 20 to 25 patients visit the ART centre daily for their medical needs. The centre was selected because of its geographical location and its capacity in serving a large number of patients from different ethnic backgrounds and various regions of Pakistan.

### Participants’ characteristics

Patients were included in the study if they fulfilled the following criteria: aged 18 years or older, diagnosed with HIV for  ≥ 6 months, have a recent CD-4 T cells count and viral load tests (no more than four weeks at the time of the interview), and can understand and converse in Urdu language (the national language of Pakistan). Only PLWHA who were taking ART on a regular basis were included in the study. Adherence to ART was confirmed by checking patients’ hospital pharmacy refill records. Prescription medical claims records can be used to estimate medication adherence based on the refill rate. Patients may routinely be defined as 'adherent patients' if the amount of medication given is at least 80% based on the medication possession ratio (MPR), which is defined as the day-to-day delivery of the medication divided by the number of days the patient should take the medication [32]. Patients with terminal illness, visual impairment, hearing impairment, non-adherence to ART, multiple comorbidities, cognitive impairment, and those unable to complete the surveys were excluded from the study.

### Sample size and sampling technique

A simple random sampling technique was used to collect data from PLWHA who fulfilled the eligibility criteria outlined above. Every PLWHA registered at the ART Centre who fulfilled the inclusion criteria had equal chance of being enrolled into the study. The sample size of 599 was calculated using the Raosoft calculator by taking a total of 180,000 HIV patients, 95% confidence interval and 4% margin of error [33]. The sample size was adjusted for unintentional error/missing rate [34] using the following formula.

$$n = \frac{n}{(1 - d)}$$

where, *n* = 599, and d = 5%. The formula provided a required sample size of 628.95 patients (*n* = 599 + n_1_ = 29.95). Overall, 630 HIV patients were approached. Out of these, 610 patients voluntarily consented to participate in the study. Six hundred and two patients were included in the final analysis.

### Study instrument and data collection procedure

EuroQol quality of life scale EQ-5D-3L and Visual Analogue Scale (VAS) have been widely used for HRQoL assessment [35]. The study protocol was registered in EuroQol Research Foundation (ID 29767) and the psychometrically validated Urdu version of EQ-5D-3L tool was obtained. EQ-5D assesses five health domains: mobility, self-care, usual activities, discomfort/pain, anxiety/depression, and each dimension is evaluated at three levels (no problem, some or moderate problem, and extreme problem). The health status of the study population can be described as 243 different health states. These health states produce a value set (index score) ranging from 1 (for the best health state, 11111) to −0.594 (for the worst health state, 33333), where a negative sign indicates QoL worse than death. EQ-5D-3L also includes a visual analogue scale (EQ-VAS), which is a vertical 20-cm calibrated line ranging from 0 to 100. The 0 describes feeble health and 100 represents the best health. The EQ-5D-3L and EQ-VAS were previously validated in Pakistan and have been used to evaluate HRQoL in patients with tuberculosis [36], hepatitis B [37], diabetes [38], and hypertension [27, 39]. The internal consistency reliability of the questionnaire (Cronbach alpha 0.72) were ensured in our study.

The data collection was carried out for a period of three months (from July 2019 to September 2019). Each person living with HIV/AIDS attending the ART centre, was approached and invited to participate in the study by the head nurse (M.J.) and HIV physician (N.B.). Those who agreed to participate were informed about the nature and purpose of the study. Each consented patient was assessed against the pre-defined inclusion and exclusion criteria by the principal investigator (A.A.). Data collection was performed at the study setting (ART Centre in PIMS Hospital). The data collection consisted of two parts: the first part covered the socio-demographic and clinical characteristics of the participants, while the second part consisted of the EQ-5D-3L and EQ-VAS. Eligible participants were asked to complete their socio-demographic characteristics (gender, age, educational level, employment status, relationship status) in Part 1 of the data collection instrument, which were confirmed from their medical records. They were also requested to self-assess their HRQoL using EQ-5D-3L and EQ-VAS in part 2 of the data collection instrument. Participants who were unable to understand any part of the questionnaire or were unable to self-administer the survey were assisted by the investigator collecting the data. The participants were instructed to read the brief instruction on the top of the page. Participants’ correct understanding was ensured, and they were encouraged to complete every question in chronological order without skipping any items. The total completion time was approximately 15–25 min. Participants’ clinical parameters, including CD4 lymphocyte count, HIV serostatus, and VL, were retrieved from the patients’ medical records. No monetary benefits or other incentives were given to study participants due to lack of funding for the study.

### Statistical analysis

Data were coded in MS Excel 2016 and imported into Statistical Package for Social Sciences (SPSS) version 21 (IBM SPSS® Statistics for Windows, version 26.0; IBM Corp, Armonk, NY, USA) for analysis. Descriptive and inferential statistics were applied as appropriate and depending on the nature of the data. Numerical variables were expressed as means and standard deviations, while categorical variables were presented as frequencies and percentages. HRQoL domains were classified as no problem, some problem and severe problem. The HRQoL utility scores (index values) were calculated using the original 1995 UK population data due to lack of utility score data from the Pakistan population [40]. Independent sample t test and One-Way ANOVA test were applied to determine the differences in HRQoL (EQ-5D index score and EQ-VAS score) by patient characteristics. Only variables responsible for significant variation (*p* < 0.05) in HRQoL in the univariate analyses were included in the regression analysis. A multivariate linear regression model was applied to find significant predictors of HRQoL among the PLWHA. Before regression analysis normal distribution of data was assessed using Shapiro–wilk test. Multivariable linear regression models with the same variables of the full and final models were ran only to assess multicollinearity using variance inflation factor diagnostics (VIF). The results are presented as unstandardized beta coefficients accompanied by 95% confidence intervals (CIs). A *p*-value of  < 0.05 was considered statistically significant.

### Ethical considerations

The ethical approval for the study was obtained from the Ethical Review Board (ERB) of ART Centre, Pakistan Institute of Medical Sciences (PIMS) and National AIDS Control Programme (NACP), Pakistan (Approval No; 1827). The objectives and procedures of the study were explained to the patients verbally. Informed consent was obtained from PLWHA who agreed to participate in the study. Moreover, potential participants were informed that participation in the study was voluntary and that they could withdraw at any time. All information collected would be kept confidential and was available to the research team only. All study procedures adopted complied with the principles of the Declaration of Helsinki, Good Clinical Practice and within the applicable laws and regulations of research involving human subjects in Pakistan.

## Results

### Sociodemographic and clinical characteristics of PLHWA in Pakistan

Of the 602 patients included in the final analysis, 65.3% (*n* = 393) were male, 41% (*n* = 247) belonged to the age group 26–50 years, and 37.7% (*n* = 227) had primary school education. Furthermore, about half (48.5%, *n* = 292) of the participants were symptomatic, 38.9% (*n* = 234) had CD 4 T cells in the range of 200–500 cells/mm^3^, more than half (51.7%, *n* = 311) had non-detectable viral load, and 64.6% (*n* = 389) got infection by injecting drugs (Table 1).

**Table 1:** Sociodemographic and clinical characteristics of PLWHA in Pakistan (*n* = 602)

Variable	*n* (%)
Gender	
Male	393 (65.3)
Female	189 (31.4)
Transgender	20 (3.3)
Age (years)	
18–25	157 (26.1)
26–50	247 (41.0)
> 50	198 (32.9)
Marital status	
Single	227 (37.7)
Married/In relationship	158 (26.2)
Divorced/separated	163 (27.1)
Widowed	54 (9.0)
Level of education	
Illiterate	173 (28.7)
Primary	227 (37.7)
Secondary	164 (27.2)
Tertiary	38 (6.3)
Employment status	
Employed	209 (34.7)
Unemployed	393 (65.3)
Duration since HIV diagnosis (years)	
< 1	181 (30.1)
1–5	237 (39.4)
6–10	120 (19.9)
> 10	64 (10.6)
HIV serostatus	
Asymptomatic	290 (48.2)
Symptomatic	292 (48.5)
AIDS-converted	20 (3.3)
CD4 count	
< 200 cells/mm^3^	190 (31.6)
200–500 cells/mm^3^	234 (38.9)
> 500 cells/mm^3^	178 (29.6)
Viral load	
Detectable	291 (48.3)
Non-detectable	311 (51.7)
Time on ART	
< 12 months	225 (37.4)
12–48 months	140 (23.3)
> 48 months	237 (39.4)
How you get infected with HIV?	
No idea	10 (1.7)
Blood products	120 (19.9)
Injecting drugs	389 (64.6)
Sex with men	50 (8.3)
Sex with women	33 (5.5)

### EQ-5D-3L scores and health states of PLHWA in Pakistan

The overall mean EQ-5D utility score was 0.388 (SD: 0.41). Of the 602 participants, more than half 56.0%, (*n* = 337) reported no impairment in mobility, 59.5% (*n* = 358) reported no impairment in self-care, while 53.5% (*n* = 322) stated no problems in usual activities. Nevertheless, 50.3% (*n* = 303) of the patients reported moderate pain/discomfort and 63.1% (*n* = 380) patients were extremely anxious/depressed. The overall mean EQ-5D 3L visual analogue scale (EQ-VAS) score was 66.20 (SD: 17.22) (Table 2).

**Table 2:** EQ-5D-3L scores of PLHWA in Pakistan (*n* = 602)

Variable	Mean (SD)
EQ-5D index score [Mean (SD)]	0.388 (0.41)
VAS score [Mean (SD)]	66.20 (17.22)
Variable	*n* (%)
Mobility	
No problem	337 (56.0)
Some problem	201 (33.4)
Confined to bed	43 (7.1)
Self-care	
No problem	358 (59.5)
Some problem	201 (33.4)
Unable to self-care	43 (7.1)
Usual activities	
No problem	322 (53.5)
Some problem	247 (41.0)
Unable to perform activities	33 (5.5)
Pain/discomfort	
No pain	256 (42.5)
Moderate pain	303 (50.3)
Extreme pain	43 (7.1)
Depression/anxiety	
Not anxious/depressed	81 (13.5)
Moderate anxious/depressed	141 (23.4)
Extremely anxious/depressed	380 (63.1)

Overall, 24 different health states were observed across the entire sample. Sixty-eight (11.3%) of the respondents reported a complete healthy state (state: 11111, index value: 1.00) with no impairment in any domain, 86 (14.3%) indicated a moderate problem in the first four domains and severe impairment in the fifth domain (state: 22223, index value: 0.082), while 23 (3.8%) reported worst health state (state: 33333, index value: −0.594) with extreme impairment in all the five domains of EQ-5D-3L (Table 3).

**Table 3:** Health states of PLHWA in Pakistan (*n* = 602)

Serial No	EQ-5D states	*n* (%)	EQ-5D index value
1	11111	68 (11.3)	1.000
2	11112	80 (13.3)	0.848
3	11113	23 (3.8)	0.414
4	11121	13 (2.2)	0.796
5	11122	37 (6.1)	0.725
6	11123	14 (2.3)	0.255
7	11212	7 (1.2)	0.812
8	11213	20 (3.3)	0.378
9	11222	17 (2.8)	0.689
10	11223	13 (2.2)	0.255
11	12113	17 (2.8)	0.310
12	12123	7 (1.2)	0.187
13	12213	21 (3.5)	0.274
14	21123	23 (3.8)	0.222
15	21213	13 (2.2)	0.309
16	21223	30 (5.0)	0.186
17	22113	7 (1.2)	0.241
18	22123	33 (5.5)	0.118
19	22223	86 (14.3)	0.082
20	22233	20 (3.3)	−0.181
21	23223	10 (1.7)	−0.028
22	23323	10 (1.7)	−0.086
23	32223	10 (1.7)	−0.163
24	33333	23 (3.8)	−0.594
	Total	602 (100.0)	

### Difference in HRQoL by patients’ characteristics

Independent sample t-test revealed that EQ-5D scores significantly differed by employment status (*P* < 0.001). One-way ANOVA test revealed that EQ-5D scores varied significantly by gender (*P* < 0.001), age (*P* < 0.001), marital status (*P* < 0.001), and level of education (*P* < 0.001). The findings indicated that asymptomatic HIV patients had significantly higher mean EQ-5D score (0.58  ±  0.37) than symptomatic HIV patients (0.26  ±  0.34) and AIDS-converted patients (−0.38  ±  0.22). In addition, HRQoL score significantly differed according to the participants’ HIV serostatus (*P* < 0.001), CD4 count (*P* < 0.001), viral load (*P* < 0.001), time to ART (*P* < 0.001), and the route of HIV infection (*P* < 0.001) (Table 4).

**Table 4:** Difference in EQ-5D scores by sample characteristics (*n* = 602)

Characteristics	EQ-5D-3L	95% CI	*P*-value	EQ-VAS	95% CI	*P*-value
	Mean ± SD			Mean ± SD		
Gender^b^			** < 0.001**			** < 0.001**
Male	0.41 ± 0.44	0.37–0.45		68.90 ± 16.78	67.24–70.57	
Female	0.29 ± 0.32	0.25–0.35		58.73 ± 15.99	56.43–61.02	
Trans gender	0.81 ± 0.03	0.80–0.83		83.50 ± 4.89	81.20–85.79	
Age^b^			** < 0.001**			** < 0.001**
18–25 years	0.63 ± 0.32	0.58–0.68		78.40 ± 14.38	76.13–80.67	
26–50 years	0.52 ± 0.33	0.48–0.56		67.61 ± 17.11	65.46–69.75	
> 50 years	0.03 ± 0.32	−0.01–0.07		54.74 ± 11.06	53.19–56.29	
Marital status^b^			** < 0.001**			** < 0.001**
Single	0.57 ± 0.39	0.52–0.62		75.06 ± 14.81	73.19–76.93	
Married/relationship	0.39 ± 0.35	0.33–0.44		64.30 ± 17.71	61.52–67.08	
Divorced/separated	0.20 ± 0.38	0.14–0.26		59.14 ± 15.49	56.74–61.53	
Widowed	0.19 ± 0.39	0.09–0.30		55.74 ± 14.74	51.71–59.76	
Level of education^b^			** < 0.001**			** < 0.001**
Illiterate	0.29 ± 0.51	0.21–0.36		62.25 ± 16.91	59.71–64.79	
Primary	0.36 ± 0.33	0.32–0.40		61.89 ± 15.49	59.86–63.92	
Secondary	0.39 ± 0.32	0.34–0.44		69.14 ± 13.44	67.07–71.22	
Tertiary	0.48 ± 0.29	0.41–0.55		97.15 ± 4.59	95.59–98.61	
Employment status^a^			** < 0.001**			** < 0.001**
Employed	0.55 ± 0.37	0.49–0.59		72.34 ± 16.69	70.06–74.62	
Non-employed	0.30 ± 0.41	0.26–0.34		62.92 ± 16.62	61.27–64.57	
Since HIV diagnosed (years)^b^			** < 0.001**			** < 0.001**
< 1	0.57 ± 0.39	0.52–0.62		74.47 ± 15.99	72.12–76.82	
1–5	0.39 ± 0.35	0.33–0.43		62.91 ± 15.90	60.87–69.94	
6–10	0.20 ± 0.38	0.14–0.26		58.25 ± 13.69	55.77–60.72	
> 10	0.19 ± 0.39	0.08–0.31		69.84 ± 20.62	64.68–75.00	
HIV serostatus^b^			** < 0.001**			** < 0.001**
Asymptomatic	0.58 ± 0.37	0.53–0.62		74.75 ± 15.27	72.99–76.52	
Symptomatic	0.26 ± 0.34	0.22–0.29		59.15 ± 15.02	57.41–60.87	
AIDS converted	−0.38 ± 0.22	−0.48–0.28		45.10 ± 5.12	42.59–47.40	
CD4 count^b^			** < 0.001**			** < 0.001**
< 200	0.03 ± 0.30	−0.09–0.07		52.21 ± 9.83	50.80–53.61	
200–500	0.34 ± 0.26	0.31–0.37		62.13 ± 12.06	60.58–63.69	
> 500	0.83 ± 0.22	0.79–0.87		86.46 ± 8.25	85.23–87.68	
Viral load^a^			** < 0.001**			** < 0.001**
Detectable	0.22 ± 0.34	0.18–0.26		58.07 ± 12.77	56.60–59.54	
Non-detectable	0.55 ± 0.41	0.50–0.59		73.79 ± 17.40	71.85–75.73	
Time on ART^b^			** < 0.001**			** < 0.001**
< 12 months	0.50 ± 0.35	0.45–0.55		72.97 ± 14.15	71.11–74.83	
12–48 months	0.56 ± 0.32	0.51–0.62		66.64 ± 18.09	63.61–69.66	
> 48 months	0.18 ± 0.41	0.13–0.23		59.49 ± 16.84	57.33–61.64	
How you get infected with HIV?^b^			** < 0.001**			** < 0.001**
No idea	0.12 ± 0.17	0.09–0.17		55.35 ± 16.49	51.15–59.55	
Blood products	0.30 ± 0.46	0.21–0.39		69.01 ± 14.63	66.35–71.64	
Injecting drugs	0.40 ± 0.41	0.3–0.44		66.45 ± 16.42	64.81–68.09	
Sex with women	0.34 ± 0.31	0.26–0.43		57.40 ± 14.11	53.38–61.41	
Sex with men	0.69 ± 0.32	0.58–0.81		68.18 ± 32.15	56.77–79.58	

Bold values show a statistically significant difference

*EQ* EuroQoL, *SD* Standard deviation, *CI* Confidence interval

^a^Independent samples *t*-test

^b^One-Way ANOVA was applied to calculate *p*-value

Similarly, VAS scores significantly differed by employment status (*P* < 0.001), gender (*P* < 0.001), age (*P* < 0.001), marital status (*P* < 0.001), and level of education (*P* < 0.001). Furthermore, asymptomatic HIV patients had the highest mean VAS score (74.75  ±  15.27) compared to symptomatic HIV patients (59.15  ±  15.02) and AIDS-converted patients (45.10  ±  5.12). Additionally, patients with CD4 count > 500 cells/mm^3^ had significantly higher VAS score (86.46  ±  8.25) than patients with CD4 count 200–500 cells/mm^3^ (62.13  ±  12.06) and CD4 count < 200 cells/mm^3^ (52.21  ±  9.83). VAS scores also significantly differed by HIV serostatus (*P* < 0.001), viral load (*P* < 0.001), time to ART (*P* < 0.001), and route of HIV infection (*P* < 0.001) (Table 4).

### Regression analysis

Standard multiple regression analysis was used to assess the ability of socio-demographic and clinical characteristics to predict HRQoL in PLWHA in Pakistan as indicated by EQ-5D index (Table 5). Preliminary analyses were conducted to ensure no violation of the assumptions of normality, linearity, multicollinearity and homoscedasticity. A good fit model for multivariate linear regression was determined (*F* = 178.82, *P* < 0.001, adjusted R^2^ = 0.881). The regression model results (unstandardized β; *p*-value) revealed that factors significantly associated with HRQoL were: female vs. male (0.118; < 0.001); age > 50 years vs. 18–25 years (0.054; 0.042); primary education vs. illiterate (−0.139; < 0.001), secondary education vs. illiterate (−0.163; < 0.001); non employed vs. employed (−0.070; < 0.001); years with HIV 1–5 years vs. < 1 year (0.289; < 0.001), 6–10 years vs. < 1 year (0.464; < 0.001), > 10 years vs. < 1 year (0.615; < 0.001); HIV serostatus symptomatic vs. asymptomatic (−0.145; < 0.001), AIDS-converted vs. asymptomatic (−0.638; < 0.001); CD4 count 200–500 cells/mm^3^ vs. < 200 cells/mm^3^ (0.173; < 0.001), > 500 cells/mm^3^ vs. < 200 cells/mm^3^ (0.670; < 0.001); viral load detectable > 20 copies/mL vs. non-detectable (−0.038; 0.041); time on ART 12–48 months vs. < 12 months (−0.338, < 0.001), > 48 months vs. < 12 months (−0.712; 0.001); no idea vs. sex with a man (−0.202; 0.001), blood products vs. sex with a man (−0.262; < 0.001).

**Table 5:** Univariate and multivariable linear regression analyses to determine the predictors of HRQoL in PLHWA in Pakistan (*n* = 602)

Patient characteristics	EQ-5D index value (Univariate linear regression)		*P*- value	EQ-5D index value (Multivariate linear regression)		*P*- value
	Unstandardized β	95% CI		Unstandardized β	95% CI	
Gender						
Female vs. male	−0.110	−0.179 to −0.0405	0.002	0.118	0.089–0.149	** < 0.001**
Transgender vs. male	0.404	0.225–0.584	< 0.001	−0.054	−0.137–0.027	0.193
Age						
26–50 years vs. 18–25 years	−0.112	−0.1763 to −0.0481	0.001	0.158	0.114–0.202	** < 0.001**
> 50 years vs. 18–25 years	−0.606	−0.673 to −0.539	< 0.001	0.054	0.002–0.106	**0.042**
Marital status						
Married/In relationship vs. single	−0.186	−0.263 to −0.109	< 0.001	0.139	0.089–0.189	** < 0.001**
Divorced/separated vs. single	−0.370	−0.447 to −0.294	< 0.001	0.006	−0.036–0.048	0.771
Widowed vs. single	−0.377	−0.490 to −0.265	< 0.001	−0.003	−0.059- 0.052	0.914
Level of education						
Primary vs. illiterate	0.0698	−0.005–0.145	0.066	−0.139	−0.183 to −0.094	** < 0.001**
Secondary vs. illiterate	0.105	0.025–0.186	0.010	−0.163	−0.211 to −0.115	** < 0.001**
Tertiary vs. illiterate	0.711	0.578–0.844	< 0.001	−0.021	−0.904–0.048	0.550
Employment status						
Non-employed vs. employed	−0.242	−0.308 to −0.176	< 0.001	−0.070	−0.104 to −0.036	** < 0.001**
HIV since diagnosis (years)						
1–5 vs. < 1	−0.125	−0.203 to −0.048	0.002	0.289	0.233–0.346	** < 0.001**
6–10 vs. < 1	−0.274	−0.367 to −0.182	< 0.001	0.464	0.380–0.548	** < 0.001**
> 10 vs. < 1	−0.123	−0.237 to −-0.009	−0.034	0.615	0.526–0.705	** < 0.001**
HIV serostatus						
Symptomatic vs. asymptomatic	−0.319	−0.377 to −0.262	< 0.001	−0.145	−0.176 to −0.111	** < 0.001**
AIDS-converted vs. asymptomatic	−0.953	−1.113 to −0.794	< 0.001	−0.638	−0.731 to −0.545	** < 0.001**
CD4 count						
200–500 vs. < 200	0.306	0.256–0.356	< 0.001	0.173	0.125–0.221	** < 0.001**
> 500 vs. < 200	0.798	0.744–0.852	< 0.001	0.670	0.604–0.739	** < 0.001**
Viral load						
Detectable vs. non-detectable	−0.334	−0.393 to −0.273	< 0.001	−0.038	−0.074 to −0.002	**0.041**
Time on ART						
12–48 months vs. < 12 months	0.062	−0.017–0.141	0.123	−0.338	−0.393 to −0.281	** < 0.001**
> 48 months vs. < 12 months	−0.320	−0.388–0.252	< 0.001	−0.712	−0.790 to −0.634	**0.001**
How you get infected with HIV?						
No idea vs. sex with men	−0.577	−0.862 to −0.293	< 0.001	−0.202	−0.323 to −0.079	**0.001**
Blood products vs. sex with men	−0.391	−0.546 to −0.237	< 0.001	−0.262	−0.329 to −0.194	** < 0.001**
Injecting drugs vs. sex with men	−0.294	−0.437 to −0.151	< 0.001	−0.037	−0.09–0.260	0.251
Sex with women vs. sex with men	−0.352	−0.529 to −0.175	< 0.001	0.211	0.139–0.282	** < 0.001**

Normality of data was assessed by QQ-plot and Shapiro–wilk test (*P* > 0.05)

Model statistics: F: 178.82; *P* < 0.001; R^2^: 0.885, Adjusted R^2^: 0.881

## Discussion

This is the first study in Pakistan that assessed the HRQoL of PLWHA who actively sought testing and receiving ART treatment. Therefore, the findings cannot be generalized to all PLWHA in Pakistan, particularly those not receiving treatment or not adherent to ART. Given the increasing incidence of HIV [1] in recent years, such a study was needed to evaluate the QoL of PLWHA in Pakistan to further develop tailored interventions. Our findings suggested that several independent factors such as male gender, transgender, young age, being employed, having higher CD-4 lymphocytes count, undetectable viral load, higher education level, and being asymptomatic were significantly associated with higher HRQoL.

The mean EuroQol 5D-3L score for PLWHA was 0.388, which could not be compared to the general population health as no data were available for Pakistan. Studies conducted in Brazil [8] and South Africa [41] reported a mean score of 0.82 and 0.80, which were higher scores than the present study. Likewise, our findings showed a score lower than the average score of 0.74 found in another study conducted in England. [30]. Regarding EQ-VAS score, our results are consistent with the South African study [41], but varied from Nglazi et al. [42] study that reported VAS median score of 90 for PLWHA adherent to ART. High level of depression is a significant predictor of lower HRQoL [43], with more than 63% of PLWHA having severe depression and anxiety in this study. Previous studies have shown that more than 10% of anxiety and depression, are key factors affecting HRQoL of PLWHA [44, 45]. Therefore, psychological interventions are needed in ART centres to improve HRQoL associated with mental health [46]. A randomized controlled trial conducted in Pakistan had reported that multi-component behavioural interventions had resulted in a significant reduction in anxiety and depression within 3 months in psychologically distressed adults [47].

In our study, older age and lower level of education were significant predictors of poor HRQoL; these results are in agreement with those obtained by Brazilian and Italian studies [8, 48]. Higher education levels may lead to a better capacity to cope with HIV, contribute to improvement of patient knowledge about the disease, and ultimately HRQoL domains [49, 50]. HRQoL decreased with an increase in age, and these results are in agreement with those obtained by American, French and Brazilian studies [8, 15, 51]. Memory issues, anxiety and depression, gender, ethnicity, economic factors, and family situation have been identified as leading factors to lower HRQoL in individuals over 50 years of age with HIV [52, 53]. It is worthwhile to explore ageing in the presence of comorbidities, frailty, anxiety, and depression to improve survival and HRQoL in PLWHA [8, 54].

Male and transgender patients with HIV had better HRQoL compared to female patients based on ANOVA test, and these results are consistent with Zimbabwean and Italian studies [48, 55]. In general, females are the most marginalized population, perhaps due to the high stigma and discrimination against them in Pakistan [56]. It can, therefore, be inferred that gender is a significant social determinant of health and that variations between gender-related HRQoL factors need to be addressed [57]. The current study found that the average HRQoL score of singles was more than married and widowed patients, which is consistent with the evidence from a previous study conducted in Ethiopia [58]. The finding that married or cohabiting people exhibiting a lower level of spiritual HRQoL may be attributed to couples' self-accusation of contracting and/or transferring the virus to one another or fear of losing their partner (due to AIDS-related causes) in the future [59]. However, some studies have reported that being in a relationship is a good predictor of quality life as it lowers the chances of loneliness and depression [8, 13, 51]. Unemployment was another predictor that resulted in lower HRQoL. This was also documented in other studies conducted in Vietnam and Japan [60, 61], because employment could be linked to higher social well-being of PLWHA. Regarding the sensitivity of EQ-5D, AIDS-converted patients demonstrated significantly lower HRQoL compared to symptomatic and asymptomatic patients, especially in physical health and pain, a finding that was also reported by other studies [62, 63]. We enrolled only adherent patients and adherence is linked to viral suppression and subsequent asymptomatic state. However, we identified AIDS-converted and symptomatic patients, which may be attributed to several factors including drug interactions and viral drug resistance [64, 65], and potentially natural disease progression.

EQ-5D VAS showed a significant relationship between HRQoL domains and CD4 lymphocytes count of  > 200 cells/mm^3^. With an increase in CD4 lymphocytes count, physical HRQoL improved. Similarly, other studies have reported CD4 lymphocytes count as an important predictor of other domains of QoL [11, 13, 66, 67]. Some studies have reported that lower CD4 count can affect mental health, possibly due to distress associated with infection progression [68, 69]. Furthermore, the present study has reaffirmed that suppression of viral load is a good predictor of HRQoL. Likewise, several other studies have predicted viral suppression to enhance physical health [18, 51] and mental health [70], and this can only be accomplished through a consistent lifelong adherence to ART [71]. Another advantage of viral suppression is that it stops the transmission of HIV to a non-HIV partner, thus supporting the worldwide slogan of Undetectable = Untransmittable [72].

### Strengths and limitations

This is the first study to assess HRQoL in PLWHA in Pakistan with a large sample size. Second, EQ-5D VAS was used to support the results of EuroQol questionnaire in its sensitivity to differentiate QoL of PLWHA based on disease stage, VL and CD-4 T lymphocytes. Third, the current findings have implications for medical decision-making to design evidence-based interventions across age groups and cultures by considering the chronic nature of HIV infection and patient reported outcomes.

The study has some potential limitations that are inherent to most observational questionnaire-based studies. First, a cross-sectional study design was used in this study; therefore, reverse causality is possible, and the findings need to be confirmed using a longitudinal study design. Second, we have not enrolled patients who failed to follow up regularly based on their dispensing records; therefore, the results cannot be generalized to non-adherent HIV-AIDS patients. These results may only be generalized to the 10% patients who are actively seeking treatment from NACP. Finally, there is a potential for social desirability bias since the respondents are likely to underreport socially undesirable and behaviors and to over report more desirable attributes.

## Conclusion

Overall, PLWHA in Pakistan who were adherent to their ART had demonstrated a good HRQoL. The EQ-5D-3L instrument was found to be sensitive in detecting QoL differences based on HIV clinical stages, number of CD-4 T lymphocytes and VL. Female gender, older age (> 50 years), low education, living with a partner/spouse, unemployed, being HIV symptomatic, AIDS-converted, lower CD 4 lymphocyte count, and detectable viral load were the factors that could predict poor HRQoL in our population and need special attention to improve HRQoL. Adherence to ART significantly improved HRQoL, except for psychological component, suggesting a need for training of healthcare professional caring for PLWHA on the screening and management of anxiety and depression among HIV patients. Psychoeducation should be conducted during initial evaluation to reduce negative beliefs regarding ART. The findings of the present study provide support for future work to design effective and holistic interventions that are culturally sensitive for Pakistan. Further studies on the determinants of health outcomes in this vulnerable population are also warranted.

## Data Availability

Data sets used in this study can be requested from the corresponding author if justified.
